# Machine learning models of tobacco susceptibility and current use among adolescents from 97 countries in the Global Youth Tobacco Survey, 2013-2017

**DOI:** 10.1371/journal.pgph.0000060

**Published:** 2021-12-08

**Authors:** Nayoung Kim, Wei-Yin Loh, Danielle E. McCarthy

**Affiliations:** 1 Center for Tobacco Research and Intervention, University of Wisconsin School of Medicine and Public Health, Madison, Wisconsin, United States of America; 2 Department of Statistics, University of Wisconsin, Madison, Wisconsin, United States of America; Jordan University of Science and Technology Faculty of Medicine, JORDAN

## Abstract

Adolescents are particularly vulnerable to tobacco initiation and escalation. Identifying factors associated with adolescent tobacco susceptibility and use can guide tobacco prevention efforts. Novel machine learning (ML) approaches efficiently identify interactive relations among factors of tobacco risks and identify high-risk subpopulations that may benefit from targeted prevention interventions. Nationally representative cross-sectional 2013–2017 Global Youth Tobacco Survey (GYTS) data from 97 countries (28 high-income and 69 low-and middle-income countries) from 342,481 adolescents aged 13–15 years (weighted N = 52,817,455) were analyzed using ML regression tree models, accounting for sampling weights. Predictors included demographics (sex, age), geography (region, country-income), and self-reported exposure to tobacco marketing, secondhand smoke, and tobacco control policies. 11.9% (95% CI 11.1%-12.6%) of tobacco-naïve adolescents were susceptible to tobacco use and 11.7% (11.0%-12.5%) of adolescents reported using any tobacco product (cigarettes, other smoked tobacco, smokeless tobacco) in the past 30 days. Regression tree models found that exposure or receptivity to tobacco industry promotions and secondhand smoke exposure predicted increased risks of susceptibility and use, while support for smoke-free air policies predicted decreased risks of tobacco susceptibility and use. Anti-tobacco school education and health warning messages on product packs predicted susceptibility or use, but their protective effects were not evident across all adolescent subgroups. Sex, region, and country-income moderated the effects of tobacco promotion and control factors on susceptibility or use, showing higher rates of susceptibility and use in males and high-income countries, Africa and the Americas (susceptibility), and Europe and Southeast Asia (use). Tobacco policy-related factors robustly predicted both tobacco susceptibility and use in global adolescents, and interacted with adolescent characteristics and other environments in complex ways that stratified adolescents based on their tobacco risk. These findings emphasize the importance of efficient ML modeling of interactions in tobacco risk prediction and suggest a role for targeted prevention strategies for high-risk adolescents.

## Introduction

Tobacco use is the leading preventable cause of noncommunicable disease (NCD) and mortality worldwide, contributing to more than 8 million premature deaths annually [1]. The tobacco epidemic continues to grow in low-and middle-income countries (LMICs) and adolescent tobacco use continues to be prevalent around the world [2, 3]. The Lancet NCD Action Group and the NCD Alliance identified tobacco control as a top-priority and cost-effective way to accelerate NCD prevention and control [4]. Recently, the United Nations (UN) urged all ratified countries to fully implement the World Health Organization (WHO) Framework Convention on Tobacco Control (FCTC), an international treaty of evidence-based tobacco control measures, to reduce the global burden of tobacco use and NCD [1]. Such tobacco control efforts have great potential to prevent NCD and improve global health.

Adolescence is a critical period of vulnerability for tobacco initiation and progression, with 90% of people who smoke beginning tobacco use in adolescence [5]. Early initiation (i.e., before age 16) of tobacco use confers risks for nicotine dependence and persistent use in adulthood [6, 7]. Tobacco use progresses through developmental stages including preparation/susceptibility, initiation, experimentation, regular smoking, and nicotine addiction [8, 9]. The first stage, susceptibility to tobacco use is defined as a lack of firm commitment against future tobacco use [8] and is a validated measure that predicts initiation of tobacco products (e.g., cigarettes, other smoked and smokeless tobacco products) [10]. Understanding early stages of tobacco susceptibility and use is critically important to efforts to prevent tobacco initiation and escalation. Identifying risk and protective factors associated with adolescent tobacco susceptibility and use could provide important targets for prevention interventions. In addition, identifying adolescent subgroups at high risk for susceptibility and use may inform allocation and targeting of prevention strategies. For example, global studies have identified greater adolescent tobacco use risks in certain regions (the Americas, Europe, Southeast Asia), national wealth categories (high-income countries (HICs)), and populations (male adolescents), suggesting that males in high-income nations in these regions may be in particular need of prevention efforts [3, 11].

Prior studies have identified environmental or contextual factors associated with tobacco susceptibility and use among adolescents. For example, exposure to tobacco industry marketing, parental smoking, and associated secondhand smoke exposure (SHS) are well-established tobacco-contextual risk factors for adolescent tobacco use [12–16]. Tobacco companies target adolescents via direct advertising in traditional media channels and indirect forms of marketing such as promotional activities and point-of-sale (POS) promotions [1, 5]. Systematic reviews and meta-analyses document consistent relations between various forms of tobacco marketing and greater tobacco susceptibility or use [13, 14]. Exposure or receptivity to tobacco promotions, such as possessing or being willing to accept promotional items bearing tobacco brand logos, significantly predicts increased susceptibility and experimentation in tobacco-naïve adolescents, and progression to regular use [13, 16]. Exposure to POS promotion (e.g., in-store advertising, prominent displays of tobacco products) is positively associated with tobacco susceptibility and use across a range of studies [14]. SHS exposure in various venues (e.g., home, indoor or outdoor public places) is also associated with increased tobacco susceptibility, use, and persistence [15]. In contrast, support for indoor or outdoor smoke-free air policies (SFPs) is associated with reduced susceptibility and use [11, 17].

These data suggest important targets for tobacco control measures. To date, studies of the effects of tobacco control measures, such as school-based education and product packaging health warning messages (HWMs), on adolescent tobacco susceptibility and use have yielded inconsistent results. Some studies found anti-tobacco school education reduced susceptibility [11], while others found null effects on tobacco use intention or use [18, 19]. A review reported that HWMs are generally effective in preventing tobacco use initiation and increasing quit attempts [20], although such protective effects were not evident in adolescents or males [21, 22]. Such moderating effects of sex or age are poorly understood, however.

Prior studies of risk or protective factors associated with adolescent susceptibility and initiation are limited by a focus on independent rather than interactive effects [11, 17, 18] and reliance on data from mostly HICs [14–16, 10]. The current study sought to advance the literature in adolescent tobacco prevention and control by applying machine learning (ML) models of main and complex interactive effects of diverse personal and contextual/environmental factors in predicting tobacco susceptibility or use among adolescents worldwide, using data from the Global Youth Tobacco Survey (GYTS). Traditional regression techniques have important limitations in identifying subpopulations at particularly high risk for undesirable outcomes, such as tobacco use, as a result of complex interactive effects across ecological levels (e.g., broad policy, school practices, home life). Traditional regression analyses are better suited to addressing questions regarding the importance of particular variables, or in predicting outcomes from a limited set of predictors. ML methods are more powerful for identifying complex interactions among variables that predict particularly high risk in some parts of the population [23]. In contrast to traditional regression, ML approaches can efficiently analyze many variables and all possible interactions among these variables simultaneously to stratify population subgroups based on similar outcomes [23, 24]. Because ML is focused on broader questions about how diverse predictors converge in a manner that maximally differentiate subgroups based on the outcomes of interest, it yields different results than do regression analyses. Instead of testing for statistical significance of regression coefficients for particular main or interactive effects, a ML regression tree model produces a decision tree that shows which predictors split the sample most efficiently based on the outcome, and do so in a hierarchical, branching manner. This enables us to identify combinations of tobacco policy-related factors (e.g., tobacco promotion, tobacco control programs), geographical factors (e.g., world region, country-income level), and demographics (e.g., sex) associated with adolescent tobacco susceptibility and use to identify distinct adolescent subgroups at high risk of tobacco susceptibility and use.

The current study was an exploratory secondary analysis of cross-sectional nationally representative GYTS data from 97 countries. The objectives of this study were (1) to estimate prevalence of susceptibility to tobacco use among tobacco-naïve adolescents, and the prevalence of current tobacco use among 13-15-year-old adolescents and (2) to apply ML regression tree models to the GYTS data to efficiently identify complex interactions among policy-related, geographic, and individual factors that differentiate global adolescents at high versus low risk of tobacco susceptibility and use. A robust ML algorithm, the Generalized, Unbiased, Interaction Detection and Estimation (GUIDE; http://pages.stat.wisc.edu/~loh/guide.html) [25] program, was used to identify best-fitting regression trees, and to assess the overall importance of each predictor in global adolescent tobacco risk prediction. Such ML-based risk modeling can help develop and design more effective, targeted prevention interventions to modify risk factors in adolescents at high risk for tobacco initiation and persistent use.

## Methods

### Data

We utilized the GYTS data collected between 2013 and 2017 in 97 countries [HICs (28 countries), LMICs (69 countries)] from 342,481 (weighted N = 52,817,455) adolescents aged 13–15 years. The most recent survey was selected for countries where the survey has been conducted more than once. Only countries with nationally representative data were included in analyses.

The GYTS is a school-based survey that collects information about tobacco use, knowledge, and attitudes among adolescents aged 13–15 years (www.who.int/tobacco/surveillance/gyts/en/). It employs a two-stage cluster sampling design, where schools are selected with a probability proportional to the student enrollment size at the first stage, and classes are randomly chosen within selected schools at the second stage. All students in classes were anonymously and voluntarily invited to complete a self-administered survey. The survey contained a set of global “core” questionnaire items common to all participating countries, and an optional questionnaire specific to each country. The current study analyzed items from the core questionnaire only to maximize the breadth and representativeness of included countries. The GYTS data are de-identified and publicly available, thus institutional review board approval was not required in accordance with 45 CFR 46. Additional methodological details and the survey protocol are available elsewhere (https://nccd.cdc.gov/GTSSDataSurveyResources/Ancillary/Documentation.aspx?SUID=1&DOCT=1).

### Outcome variables

The two outcomes analyzed were (1) susceptibility to tobacco use among tobacco-naïve adolescents, and (2) current tobacco use among adolescents. Adolescents were asked if they ever tried cigarettes, smoked tobacco products other than cigarettes (e.g., cigar, pipes, bidis, water pipe/hookah), or smokeless tobacco products (e.g., snuff, snus). Participants who responded “no” for all products, indicating never trying any tobacco product, were coded as tobacco-naïve. Tobacco susceptibility was measured using validated questions [8] assessing intentions to use any form of tobacco products during the next 12 months and intentions to use if one of the best friends offered them a tobacco product, with four responses (definitely not, probably not, probably yes, and definitely yes). Those who responded “definitely not” to both questions were coded as non-susceptible and the rest were coded as susceptible. A total of 206,726 (weighted N = 35,388,004) adolescents were identified as tobacco-naïve and included in models of susceptibility.

For current tobacco use, adolescents reporting any use of cigarettes, smoked tobacco products other than cigarettes, or smokeless tobacco products within the past 30 days were coded as current tobacco users. Data on specific smokeless or smoked tobacco products other than cigarettes (e.g., waterpipe) were not available in the core questionnaire administered in all 97 countries and were therefore unavailable for the current analyses. A total of 342,481 respondents (weighted N = 52,817,455) were included in models of current tobacco use. Only cases with complete outcome variables were included in analyses.

#### Predictor variables

Predictors hypothesized to be associated with susceptibility or current tobacco use were identified *a priori* based on previous research [3, 11–16, 19]. Predictor variables in the GYTS included self-reported sex (male or female), age, exposure to SHS inside home, exposure to SHS indoor public places, exposure to SHS outdoor public places, exposure to SHS inside or outside school, knowledge about harmful effects of SHS, support for indoor SFP, support for outdoor SFP, exposure to anti-tobacco media messages, exposure to anti-tobacco messages at social events, exposure to HWMs on cigarette packages, exposure to anti-tobacco school education, exposure to pro-tobacco media advertisements, and exposure to POS advertisements or promotions. Except for age, all these predictors were assessed with single categorical items and coded as binary. In addition, exposure or receptivity to tobacco industry promotions was assessed using three items [16]. This predictor was coded as binary such that endorsement of at least one item (having an item with a tobacco brand logo, ever being offered a free tobacco product, or being willing to use or wear an item with a tobacco brand logo or label on it) indicated exposure or receptivity to tobacco industry promotions (henceforth labeled exposure/receptivity). Detailed coding methods for the GYTS items are available Table A in S1 Table. Other predictors suggested by prior research (e.g., parental smoking [12]) were not included, as they were not available in the most recent waves of surveys across all included countries. In addition, given significant disparities in the prevalence of adolescent tobacco susceptibility and use across countries [1, 11], WHO geographical region and country-income level were included as predictor variables of tobacco susceptibility and use. Geographical region was coded using WHO criteria as Africa, East Mediterranean, Europe, the Americas, Southeast Asia, or Western Pacific (https://www.who.int/healthinfo/global_burden_disease/definition_regions/en/). Countries were classified as HICs or LMICs based on the World Bank classification (https://datahelpdesk.worldbank.org/knowledgebase/articles/906519-world-bank-country-and-lending-groups).

### Statistical analysis

Univariate analyses separately estimated weighted rates of tobacco susceptibility and use in each WHO region and country-income level. Models accounted for the complex survey design of the GYTS and were run with SAS 9.4 software (SAS Institute, Cary, NC).

GUIDE [25] ML algorithm for piecewise constant weighted least-squares regression trees was used. This approach to ML modeled predictors of tobacco susceptibility and use (separately), and classified subgroups of adolescents at high risk for these two outcomes. GUIDE regression tree modeling was selected based on its advantages over other decision tree modeling algorithms (e.g., CART [26]). GUIDE creates regression trees that have less selection bias than other alternative methods. GUIDE also treats missing values in predictor variables as a separate informative category and therefore does not require listwise deletion of cases with missing values, or imputation of missing values [27]. In addition, GUIDE generates importance scores that reflect the overall (main and interactive) effects of each predictor variable of interest in outcome prediction. Importance scores distinguish important predictor variables from unimportant ones with a threshold of 1.0, with scores at or above this threshold considered important [28].

GUIDE performs binary recursive partitioning to construct a hierarchical decision tree composed of unique combinations of predictors that stratify subgroups of a population (e.g., global adolescents) based on the defined outcomes (e.g., tobacco susceptibility or current use). At each partitioning step, GUIDE conducts a chi-square test to assess associations between each predictor and the outcome, and selects the most important predictor (tree split) and optimal cutoff of the predictor to subdivide the data into homogenous subgroups. The partitioning procedure enables the assessment of important predictors of tobacco susceptibility or use and identifies complex interactions among predictors that maximally differentiate adolescent subgroups at high versus low risk for tobacco susceptibility or use. GUIDE uses a cross-validation based pruning method to avoid under- or over-fitting the regression trees. In the current study, the minimum sample size is 1033 cases for tobacco susceptibility and 1712 cases for tobacco use, and a maximum of 30 split levels. Ten-fold cross-validation was used to assess predictive performance of each subtree, and the optimal final tree was then selected based on the lowest cross-validation error rates. GUIDE models used the GYTS complex sampling survey weights to generate weighted rates of susceptibility or use in separate regression trees.

## Results

### Estimates of susceptibility and current use

As shown in Table 1, overall, 12% of tobacco-naïve adolescents were susceptible to tobacco use, and another 12% of adolescents reported using any tobacco product in the past 30 days. Susceptibility and use varied significantly by WHO region, with susceptibility most prevalent in Africa and the Americas, and current use most prevalent in Europe and Southeast Asia (*ps*<0.0001). In addition, HICs had significantly higher rates of susceptibility and use than did LMICs (*ps*<0.05). Country-specific prevalence estimates of tobacco susceptibility and use, and prevalence estimates by types of tobacco product use (cigarettes, smoked tobacco products other than cigarettes, and smokeless tobacco products) in each WHO region and in each country-income level can be found in the Tables B and C in S1 Table, Figs A and B in S1 Fig.

**Table 1:** Prevalence of susceptibility to tobacco use among tobacco-naïve adolescents, and use of any tobacco product in the past 30 days among adolescents, aged 13–15 years, from 97 countries in the Global Youth Tobacco Survey, 2013–2017, by WHO region and country-income level.

	Susceptibility to tobacco use among tobacco-naïve adolescents		Current tobacco use (used any product in past 30 days)	
Region and income groups	% (N)^a^	95% CI	% (N)^a^	95% CI
WHO region				
Africa (n = 14)	16.1 (748,868)	14.3–17.9	8.1 (509,843)	7.1–9.0
The Americas (n = 20)	17.9 (569,403)	16.8–19.0	10.2 (492,295)	9.2–11.2
Eastern Mediterranean (n = 13)	12.0 (688,877)	10.0–14.1	11.3 (974,298)	9.6–12.9
Europe (n = 28)	12.5 (585,976)	11.9–13.1	14.3 (1,206,093)	13.7–14.8
Southeast Asia (n = 7)	8.6 (1,032,855)	7.0–10.1	13.0 (2,246,408)	11.1–14.9
Western Pacific (n = 15)	11.4 (573,874)	10.5–12.3	10.5 (764,118)	8.8–12.2
Across WHO region χ^2^	1860.8***		1196.9***	
Country-income				
High-income (n = 28)	16.5 (416,187)	15.6–17.5	13.0 (520,444)	12.0–13.9
Low-and middle-income (n = 69)	11.5 (3,783,666)	10.7–12.3	11.6 (5,672,611)	10.8–12.4
Across country-income χ^2^	328.3***		41.4*	
*Total (n = 97)*	11.9 (4,199,853)	11.1–12.6	11.7 (6,193,054)	11.0–12.5

^a^ Weighted percentage and weighted counts in parentheses. CI = confidence interval.

***p<0.0001

*p<0.05.

Table 2 displays characteristics of the full and analyzed samples by susceptibility and current use. Approximately half of 13-15-year-old adolescents were exposed to SHS at public places and school. Two-thirds received anti-tobacco messages via media, school, and pack warnings whereas at least one-third were exposed to POS and tobacco industry promotions. These characteristics varied by tobacco susceptibility and use status. Susceptible adolescents showed significantly higher rates of exposure to SHS in various places and tobacco promotions, but lower rates of SFPs support than did non-susceptible adolescents (*p*s<0.0001). Roughly 70% of current tobacco users reported being exposed to SHS inside or outside their homes (e.g., public places, school), but most (62%-73%) supported indoor or outdoor SFPs. At least 50% of them were exposed to diverse forms of tobacco promotions and advertisements.

**Table 2:** Sample characteristics of the full sample adolescents aged 13–15 years from 97 countries in the Global Youth Tobacco Survey, 2013–2017, and of those who currently use tobacco, tobacco-naïve adolescents who were susceptible to tobacco use, and tobacco-naïve adolescents who were not susceptible to tobacco use.

	Total sample^a^ (n = 342,481)	Current tobacco use (n = 44,099)	Tobacco-naïve adolescents susceptible to tobacco use (n = 28,711)	Tobacco-naïve adolescents not susceptible to tobacco use (n = 178,015)
Variable	% (95% CI)	% (95% CI)	% (95% CI)	% (95% CI)
Male	52.4 (50.3–54.5)	73.1 (70.7–75.4)	52.7 (49.3–56.1)	45.0 (42.5–47.5)
Age				
13 years	36.8 (35.1–38.5)	27.8 (25.1–30.5)	37.3 (34.5–40.1)	40.5 (38.9–42.2)
14 years	36.5 (35.3–37.7)	37.2 (35.0–39.5)	36.9 (34.6–39.1)	35.9 (34.4–37.3)
15 years	26.7 (25.3–28.1)	35.0 (32.4–37.6)	25.9 (23.8–28.0)	23.6 (22.2–25.0)
Exposure to SHS inside home	34.1 (32.6–35.5)	59.9 (57.1–62.6)	32.1 (30.0–34.2)	26.4 (25.0–27.8)
Exposure to SHS indoor public places	48.5 (47.1–49.8)	68.5 (66.4–70.6)	47.7 (45.4–50.1)	42.7 (41.1–44.2)
Exposure to SHS outdoor public places	50.6 (49.3–52.0)	70.1 (67.8–72.5)	49.3 (47.2–51.4)	45.1 (43.7–46.6)
Exposure to SHS inside or outside school	49.1 (47.5–50.6)	65.7 (63.3–68.0)	46.2 (43.4–49.1)	44.0 (42.4–45.6)
Knowledge about harmful effects of SHS	91.9 (90.3–91.7)	89.1 (88.0–90.2)	89.3 (87.9–90.6)	92.3 (91.4–93.2)
Support for indoor SFP	79.7 (78.3–81.1)	73.1 (70.8–75.4)	72.0 (69.5–74.5)	82.1 (80.5–83.6)
Support for outdoor SFP	74.9 (73.6–76.1)	62.1 (59.8–64.5)	65.8 (63.5–68.1)	79.3 (77.9–80.7)
Exposure to anti-tobacco media messages	64.9 (63.6–66.2)	64.9 (62.5–67.3)	62.2 (60.2–64.2)	66.0 (64.5–67.5)
Exposure to anti-tobacco messages at social events	28.0 (26.9–29.2)	35.4 (33.4–37.4)	25.9 (23.9–27.9)	27.0 (25.7–28.4)
Exposure to HWMs on cigarette packages	59.5 (58.3–60.8)	79.1 (77.8–80.5)	58.2 (55.7–60.6)	53.0 (51.4–54.5)
Exposure to anti-tobacco school education	60.1 (58.6–61.6)	59.3 (57.0–61.5)	53.1 (50.9–55.3)	61.9 (60.1–63.7)
Exposure to pro-tobacco media advertisements	59.6 (58.4–60.9)	64.4 (62.5–66.3)	57.7 (55.1–60.3)	57.5 (56.0–59.0)
Exposure to POS pro-tobacco advertisements or promotions	33.2 (31.8–34.5)	46.3 (44.0–48.7)	35.3 (33.0–37.6)	29.2 (27.7–30.8)
Exposure/receptivity to tobacco industry promotions	30.3 (29.3–31.4)	51.8 (49.2–54.3)	43.1 (41.2–45.1)	20.8 (19.7–21.8)

^a^ Weighted N = 52,817,455. n represents unweighted counts and % is weighted percentages. CI = confidence interval, SHS = secondhand smoke, SFP = smoke-free air policy, HWMs = health warning messages, POS = point-of-sale. Chi-square tests were conducted to examine differences in sample characteristics across current tobacco users versus tobacco-naïve adolescents susceptible to tobacco use and across tobacco-naïve adolescents susceptible to tobacco use versus those non-susceptible to tobacco use. All characteristics were statistically significantly different across the analyzed sample groups (*ps*<0.0001), except for exposure to anti-tobacco messages at social events across those susceptible to use versus those non-susceptible to use (p = 0.1092).

### Regression trees

#### Susceptibility to tobacco use

**Fig 1:**
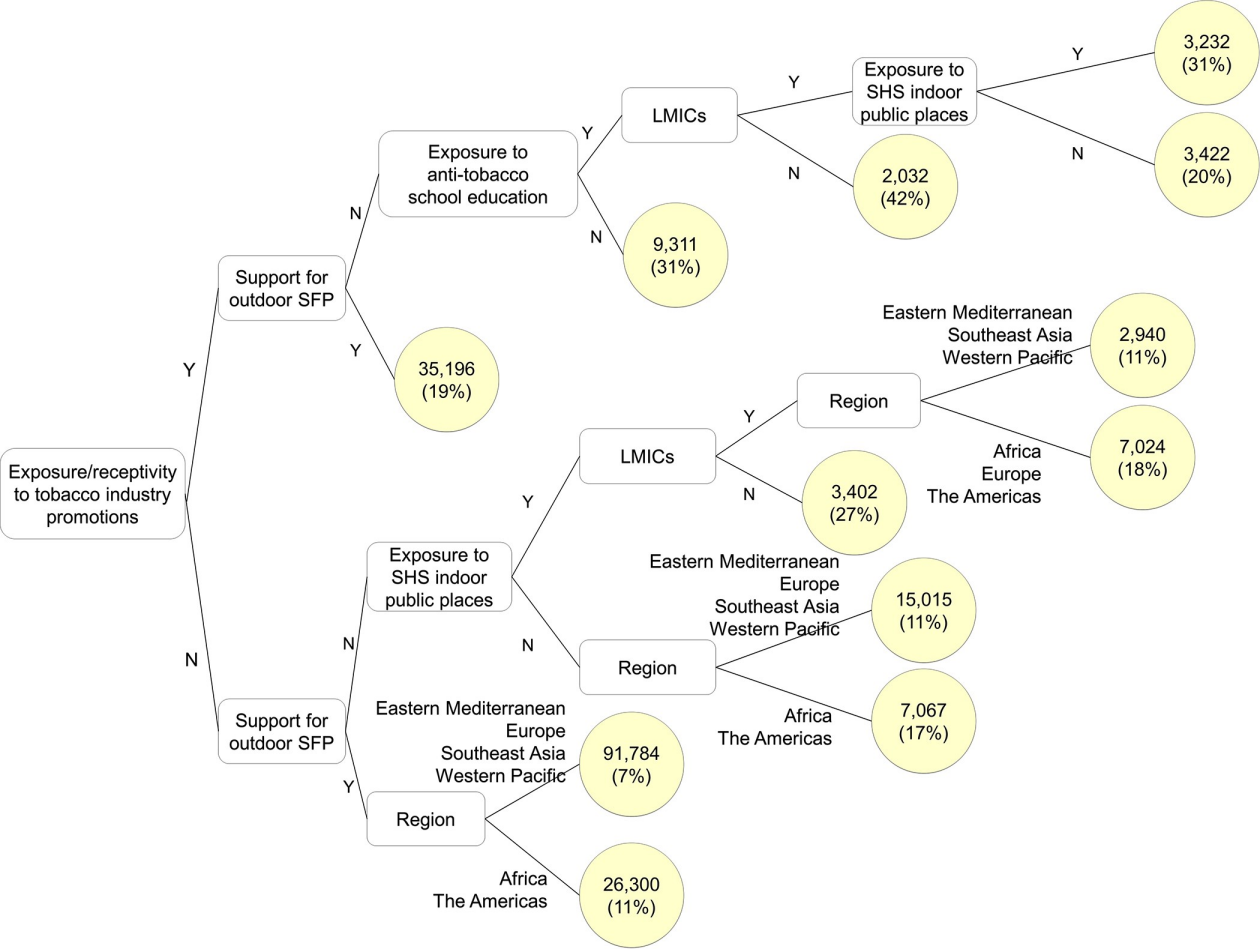
GUIDE regression tree model predicting susceptibility among tobacco-naïve adolescents aged 13–15 years from 97 countries in the Global Youth Tobacco Survey, 2013–2017. Unweighted counts of susceptibility (and weighted percentages) among subgroups are presented in yellow ovals. Y = yes, N = no, SFP = smoke-free air policy, SHS = secondhand smoke, LMICs = low-and middle-income countries. Region indicates WHO region.

The final regression tree for susceptibility among tobacco-naïve adolescents is shown in Fig 1. It identifies the optimal splits that best differentiate those susceptible from those unsusceptible to future tobacco use. The first split (by tobacco industry promotion exposure/receptivity) occurs at the left of the tree and is on the predictor that best differentiates subgroups in the higher versus lower branches of the tree. Subsequent splits illustrate the optimal splits among subgroups defined based on exposure/receptivity to tobacco industry promotions. As such, different splits in the upper versus lower branches of the tree indicate interactive effects (i.e., predictor relations with susceptibility conditional on predictor variables used in earlier splits). In this model, tobacco industry promotion exposure/receptivity was the first split (the variable that best differentiated levels of susceptibility risk overall), and stratified subgroups with rates of susceptibility ranging from 7% to 27% in those who denied exposure/receptivity to tobacco promotions (as shown in the yellow ovals at the ends of the lower branches of the tree) and from 19% to 42% in those who endorsed promotion exposure/receptivity (the upper branches of the tree). Among those exposed/receptive to promotions, those who supported outdoor SFP showed lower rates of susceptibility (19%), while those who did not support outdoor SFP had higher rates of susceptibility, especially in HIC adolescents who received anti-tobacco school education (42%) and those exposed to SHS indoor public places in LMICs (31%).

In the lower branches representing those not exposed/receptive to tobacco industry promotions, support for outdoor SFP was also associated with low rates of susceptibility, particularly in regions other than Africa and the Americas (7%). Among those who did not support outdoor SFP, exposure to SHS in indoor public places was associated with elevated risks of susceptibility in HIC adolescents (27%) and LMIC adolescents in Africa, Europe and the Americas (18%). Indeed, among those not exposed/receptive to tobacco promotions, the only group whose susceptibility exceeded that of promotion exposed/receptive adolescents was those in HICs exposed to indoor SHS who did not support SFPs.

As shown in Table 3, exposure/receptivity to tobacco industry promotions predicted susceptibility with the highest importance score. This indicates that tobacco industry promotion is very important in differentiating susceptibility subgroups across all possible splits, not just the first one. Support for SFPs, region, exposure to anti-tobacco programs via media or school, exposure to SHS in various venues, and exposure to POS promotions also had high importance scores.

**Table 3:** Ranking of regression tree predictors of tobacco susceptibility and use by importance scores.

	Susceptibility to tobacco use among tobacco-naïve adolescents		Current tobacco use (any tobacco product in the last 30 days)	
Rank	Predictor	Importance score	Predictor	Importance score
1	Exposure/receptivity to tobacco industry promotions	1050.6	Support for outdoor SFP	1143.2
2	Support for outdoor SFP	422.5	Exposure/receptivity to tobacco industry promotions	1035.9
3	Support for indoor SFP	216.3	Exposure to SHS outdoor public places	882.9
4	WHO region	175.9	Exposure to SHS indoor public places	864.9
5	Exposure to anti-tobacco media messages	112.0	Exposure to HWMs on cigarette packages	710.6
6	Exposure to anti-tobacco school education	102.6	Exposure to SHS inside home	623.9
7	Exposure to SHS outdoor public places	93.2	Exposure to SHS inside or outside school	539.7
8	Exposure to POS pro-tobacco advertisements or promotions	92.0	Support for indoor SFP	419.9
9	Exposure to SHS indoor public places	85.0	Sex	340.1
10	Country-income	75.7	Exposure to POS pro-tobacco advertisements or promotions	255.4
11	Exposure to SHS inside or outside school	64.8	Exposure to anti-tobacco media messages	216.5
12	Exposure to HWMs on cigarette packages	57.6	Exposure to pro-tobacco media advertisements	154.2
13	Exposure to SHS inside home	49.5	Exposure to anti-tobacco messages at social events	146.1
14	Knowledge about harmful effects of SHS	37.9	Knowledge about harmful effects of SHS	136.3
15	Exposure to pro-tobacco media advertisements	33.6	Exposure to anti-tobacco school education	113.1
16	Exposure to anti-tobacco messages at social events	17.2	WHO region	73.6
17	Age	9.7	Country-income	64.9
18	Sex	7.0	Age	11.3

SFP = smoke-free air policy, SHS = secondhand smoke, POS = point-of-sale, HWMs = health warning messages.

### Current tobacco use

**Fig 2:**
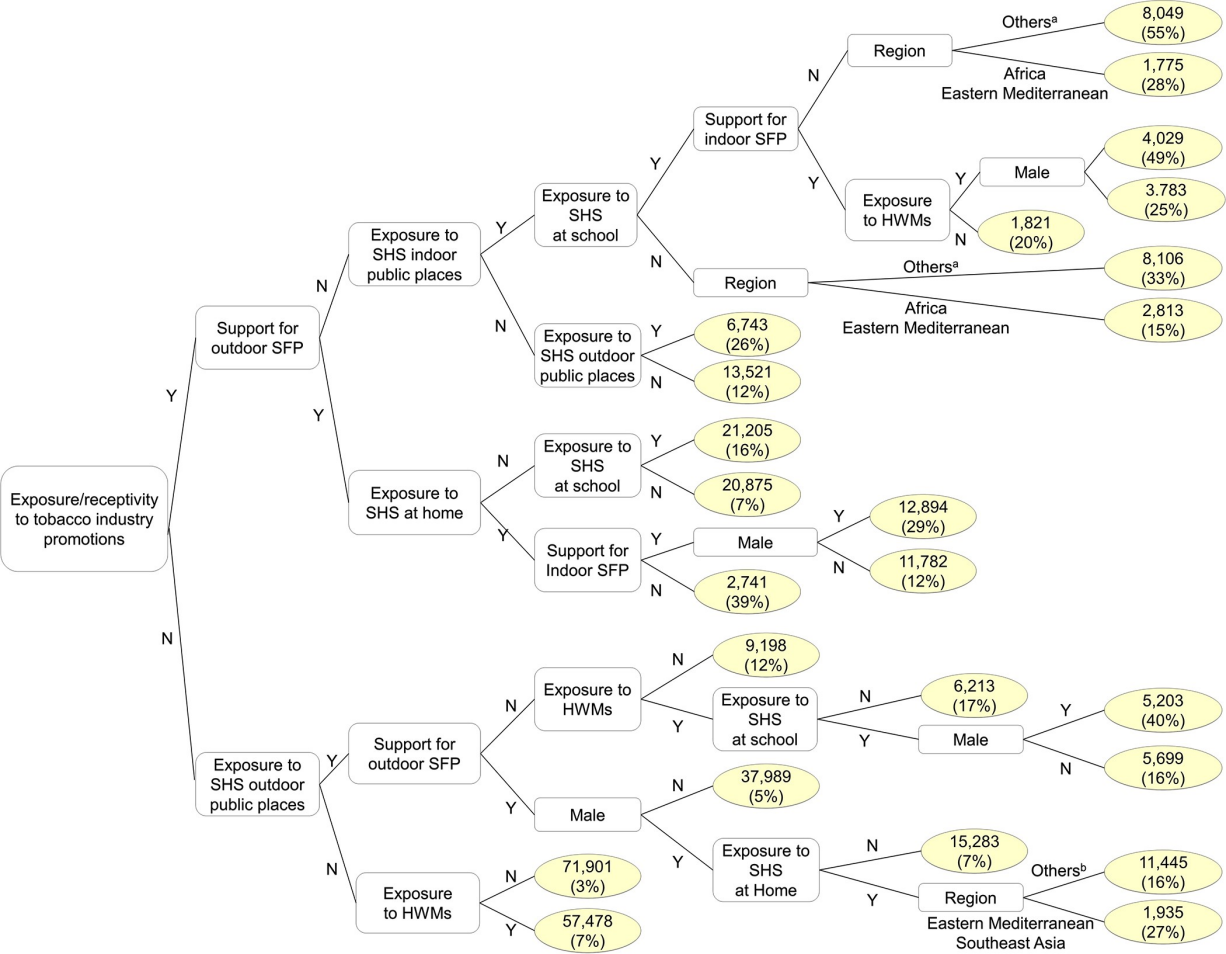
GUIDE regression tree model predicting current tobacco use among adolescents aged 13–15 years from 97 countries in the Global Youth Tobacco Survey, 2013–2017. Unweighted counts of current tobacco use (and weighted percentages) among subgroups are presented in yellow ovals. Y = yes, N = no, SFP = smoke-free air policy, SHS = secondhand smoke, HWMs = health warning messages. Region indicates WHO region. Others^a^ includes the Americas, Europe, Southeast Asia, and Western Pacific. Others^b^ includes Africa, the Americas, Europe, and Western Pacific.

As shown in Fig 2, exposure/receptivity to tobacco industry promotions was the first split in the regression tree of current tobacco use, showing relatively higher use rates among those exposed/receptive to promotions (7%-55%) than those not exposed/receptive (3%-40%). In the upper branches of the tree (those exposed/receptive to promotions), support for outdoor SFP emerged as the second split, such that endorsing SFP was associated with decreased rates of use. Complex interactions with SHS exposure, HWM exposure, support for indoor SFP, sex, and region also emerged. Particularly high rates of use were observed in those exposed to SHS in schools and indoor public places who did not support SFPs and lived in regions other than Africa and Eastern Mediterranean (55%), and in males exposed to HWMs (and therefore to tobacco products bearing the HWMs) who were also exposed to SHS in school and public places, even when they supported indoor SFP (49%). Even with support for outdoor SFP, use rates were quite high (39%) among those with home SHS exposure and those against indoor SFP.

In the lower branches of the tree (those not exposed/receptive to tobacco industry promotions), not being exposed to SHS outdoor public places or to HWMs, support for outdoor SFP, and being female were associated with low rates of current use (3%-7%). In contrast, high rates of use were found in males exposed to SHS in schools and to HWMs who were not supportive of outdoor SFP (40%) and males residing in Eastern Mediterranean or Southeast Asia regions who were exposed to SHS at home (27%).

Table 3 shows that exposure/receptivity to promotions and support for outdoor SFP had the highest importance scores in tobacco use models. Exposure to SHS in varying places, HWM exposure, support for indoor SFP, and sex also showed high importance scores.

## Discussion

Efficient ML regression tree models with representative data from 97 countries identified exposure/receptivity to tobacco industry promotions, SHS exposure in various venues, and support for SFPs as important predictors of susceptibility to tobacco use among tobacco-naïve adolescents, and of tobacco use in the past 30 days among 13-15-year-olds. These targets of tobacco control polices/measures interacted with one another and with sex, region, and country-income in ways that differentiate risk of tobacco susceptibility and use.

Exposure/receptivity to tobacco industry promotions was endorsed by 30%-50% of the GYTS respondents and was strongly predictive of both tobacco susceptibility and past-30-day use. This is consistent with prior studies showing strong relations between tobacco promotions and adolescent tobacco use intention and progression [13, 16]. Exposure to POS tobacco promotions was also predictive of susceptibility and use, as indicated by high importance scores across all trees, although this exposure was not retained in the final regression trees. Banning indirect tobacco marketing has been effective in mitigating attractiveness of tobacco products and reducing tobacco initiation and consumption in young people [29, 30]. Despite evidence of the effectiveness of tobacco control policies limiting promotions and advertisements, only 18% of the world population is currently covered by comprehensive tobacco marketing and promotion bans [1]. The current findings suggest that exposure/receptivity to tobacco promotions are still common in global adolescents and potentially consequential in their tobacco use risk. High-level compliance and implementation of comprehensive bans on diverse forms of tobacco marketing (WHO’s FCTC Article 13) may effectively redress this persistent issue [31]. In addition, high importance scores from the current study suggest that exposure to anti-tobacco media messages is predictive of lower risk of tobacco susceptibility and use. This finding adds to data suggesting that strengthening tobacco control advertising may be an effective public health intervention to mitigate risks of initiation of tobacco use and progression to more intensive use [32].

Exposure to SHS at home, school or public places also predicted tobacco susceptibility and use, consistent with prior studies [15]. Tobacco susceptibility and use rates were higher among those exposed to SHS than among those not exposed, at both levels of tobacco industry promotion exposure/receptivity (i.e., in all major branches of the regression tree models). Exposure to SHS had high importance scores across all trees, as well. These findings are consistent with evidence that SFPs enforcement in various venues (including homes and schools) help prevent and curb tobacco use among adolescents at population-level [33–35].

Support for SFPs strongly predicted decreased susceptibility and use. The vast majority of the GYTS respondents supported SFPs banning smoking in public places. In the regression trees of both susceptibility and use, support for outdoor SFP was the second split and had very high importance scores. Support for SFPs counteracted risks of exposure/receptivity to promotions and SHS exposure in various places in predicting susceptibility and use (unless this support did not extend to indoor SFP for those with SHS at home). These findings support evidence that campaigns to raise support for SFPs can be highly effective for tobacco prevention, and support the importance of home SFPs [17, 35].

School-based anti-tobacco education was associated with lower tobacco susceptibility (but not use) in those exposed to tobacco promotions, but in limited situations (e.g., in adolescents from LMICs who did not support outdoor SFP and were not exposed to SHS indoors). Exposure to HWMs was associated with higher rates of current tobacco use (but not susceptibility), particularly for males. This positive relation between HWMs and current tobacco use may reflect greater exposure to tobacco packaging due to ongoing use (i.e., reverse causality) or greater exposure to others’ smoking (i.e., a third variable influencing both use and HWM exposure). As such, robust protective effects of anti-tobacco education and HWMs were not evident across adolescent subgroups in the final regression trees, but high importance scores suggest that these tobacco control policies are associated with susceptibility and use.

Sex, region, and country-income were predictive of tobacco susceptibility and use, but their importance scores were relatively low. Such factors moderated the effects of tobacco control policy-related contextual factors on susceptibility or use, as shown in the branches near the terminal nodes of the regression trees. Within subgroups based on exposure/receptivity to tobacco promotions, exposure to SHS and support for SFPs, differences in rates of susceptibility or use by sex, region, and country-income emerged. These effects were consistent with the overall population findings of greater susceptibility and use among males and HICs, greater susceptibility in Africa and the Americas, and greater use in Europe and Southeast Asia, but were more pronounced in some specific branches of the regression trees. These findings suggest that, although policy-related contextual factors have fairly robust associations with susceptibility and use, these are not uniform across subpopulations defined by the intersections of sex, region and country-income. These complex moderation effects highlight the value of efficient ML approaches that can examine complex interactions across ecological levels of analysis and identify subgroups at particularly high risk for tobacco use. Recognition of these variations in tobacco control relations with adolescent tobacco use may facilitate refinement and targeting of tobacco control measures to reach these high-risk groups.

Although this study has several strengths including the use of representative data from 97 countries and an unbiased ML regression tree approach, it also has limitations. First, cross-sectional data cannot establish causation or casual priority. Second, policy contexts were assessed via adolescent self-report rather than via a formal policy analysis. Thus, observed associations do not necessarily demonstrate actual effects of tobacco control policy/measure implementation, and instead reflect associations among adolescent behaviors, attitudes, and awareness of tobacco promotion and control activities. Third, we included only school-attending adolescents aged 13–15 years to be in consistent with the GYTS sampling frame. This limited age range limits the generalizability of findings to other groups of adolescents. Fourth, we limited analysis to variables from the most recent version of the GYTS core questionnaire common to all participating countries to maximize inclusion and enhance global representation. Limiting analyses to items in the core questionnaire precluded modeling of other important predictors (e.g., parental tobacco use), outcomes (e.g., dependence) or modeling risk of use of specific tobacco products (e.g., waterpipe, electronic cigarettes). We instead examined broader predictors (e.g., SHS exposure from anyone in the home [36]) and outcomes (e.g., use of any tobacco product). As such, the study is unable to address regional differences in susceptibility to and use of specific tobacco products (e.g., waterpipe use in Eastern Mediterranean nations). Supplementary analyses instead showed that prevalence of currently using smoked tobacco products other than cigarette was most prevalent in the Eastern Mediterranean (4.4%) and European (7.8%) regions. Fifth, although we considered GUIDE the best ML method for the current study, other regression tree-based ensemble methods (e.g., Random Forest and Extreme Gradient Boosted Trees [24, 37]) can also identify predictive models of tobacco use. Hybrid ML approaches that combine multiple regression tree algorithms may yield additional information and improve model accuracy. Finally, all predictors (except for age) and outcomes were treated as categorical, and this approach may mask dose-dependent associations (e.g., repeated exposure to promotions and increased likelihood of susceptibility) with tobacco susceptibility and use.

In conclusion, this study identified factors across ecological levels that are associated with early adolescent tobacco susceptibility and use around the world, many of which can be influenced by tobacco control measures. ML models efficiently identified that tobacco promotion exposure, SHS exposure and support for SFPs interacted with one another and with sex, region, and national wealth in predicting differential risks of global adolescent susceptibility and use. Results are consistent with recommended comprehensive implementation of WHO FCTC control measures (e.g., regulation of tobacco promotion, smoke-free policies, and anti-tobacco education and messages) and identify subpopulations that may benefit from targeted tobacco prevention interventions for adolescents at particularly high risk for future and persistent tobacco use. Findings highlight the need to regulate tobacco industry promotions, enforce SFPs, and prevent SHS (across private, public, indoor, and outdoor spaces), as all these modifiable risk factors predict increased risk of tobacco use in global adolescents. Anti-tobacco messaging via diverse media channels and gender- and age-targeted prevention campaigns may mitigate risks for high-risk adolescent subgroups. This application of ML methods to global data also highlights the value of using “big data” analytic approaches to advance health policy knowledge and prevention interventions in tobacco control and public health [38, 39]. Use of the integrated big data (e.g., surveillance data, medical health records) and ML help screen population and predict tobacco use risk and its associated diseases at adolescent population-level. Such approaches also may help refine tobacco control measures and policies and inform allocation of resources targeting adolescent subgroups in particular need of tobacco prevention and control.

## Supporting information

S1 Fig (DOCX)

S1 Table (DOCX)
