# Supplementary material for: Machine learning models of tobacco susceptibility and current use among adolescents from 97 countries in the Global Youth Tobacco Survey, 2013-2017
Source: PLOS Glob Public Health. 2021 Dec 8;1(12):e0000060. doi: 10.1371/journal.pgph.0000060 (PMC10021689; doi:10.1371/journal.pgph.0000060)

**S1 Fig**

**Figure A. Country-specific prevalence estimates (%) of susceptibility to a tobacco use among tobacco-naïve adolescents, aged 13-15 years, from 97 countries in the Global Youth Tobacco Survey, 2013-2017.**

CI=confidence interval. In the parentheses next to country name, the first code indicates country-income level category (LM=low- and middle-income, H=high-income) and the second code indicates WHO region category (AF=Africa, AM=The Americas, EM=Eastern Mediterranean, EU=Europe, SA=Southeast Asia, WP=Western Pacific). FYR Macedonia refers to the former Yugoslav Republic of Macedonia and Laos refers to the Lao People's Democratic Republic The prevalence estimate of susceptibility in Bahamas may not represent an accurate population estimate of susceptibility due to a high standard error.


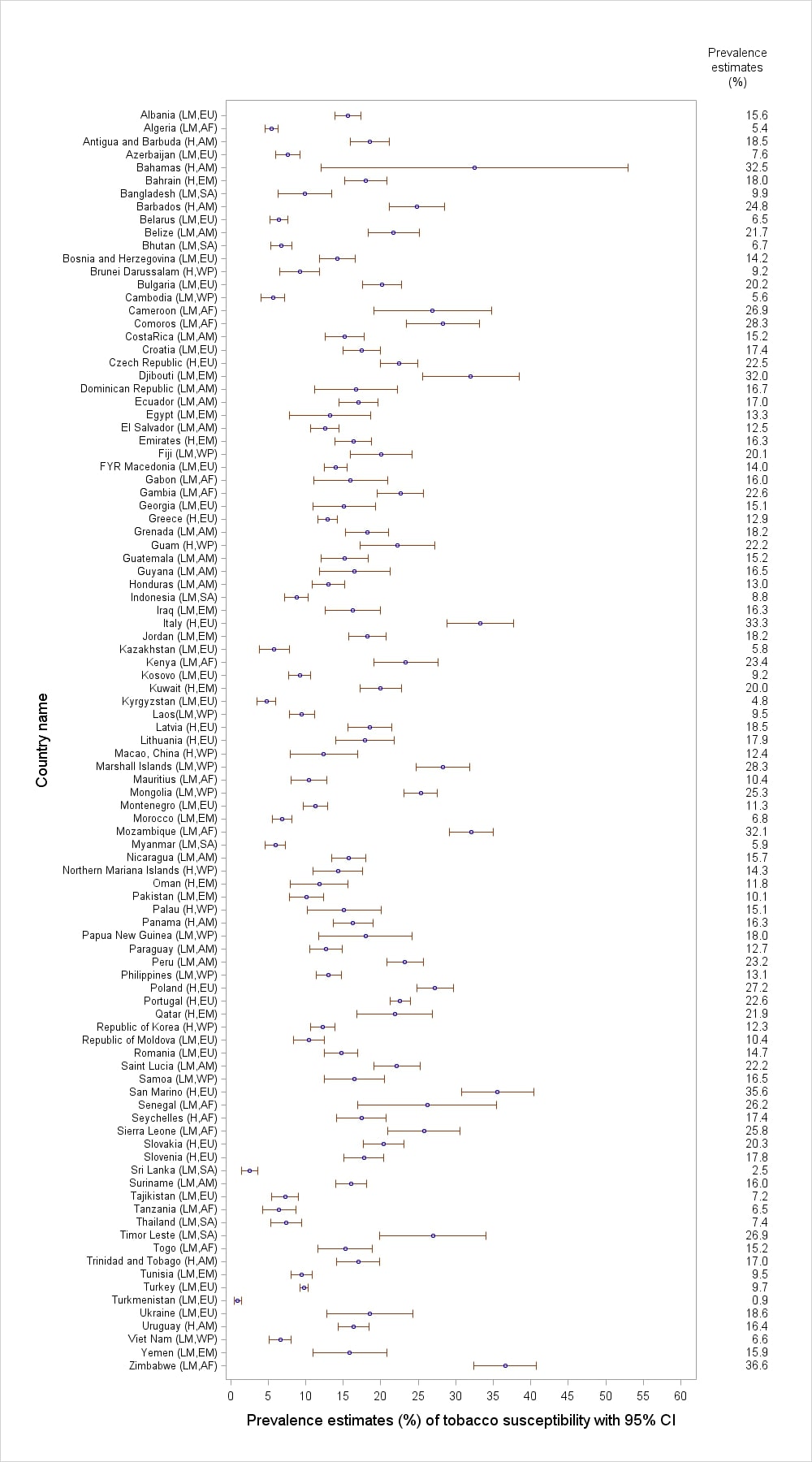


**Figure B. Country-specific prevalence estimates (%) of use of any tobacco product in the past 30 days among adolescents, aged 13-15 years, from 97 countries in the Global Youth Tobacco Survey, 2013-2017.**

CI=confidence interval. In the parentheses next to country name, the first code indicates country-income level category (LM=Low- and middle-income, H=High-income) and the second code indicates WHO region category (AF=Africa, AM=The Americas, EM=Eastern Mediterranean, EU=Europe, SA=Southeast Asia, WP=Western Pacific). FYR Macedonia refers to the former Yugoslav Republic of Macedonia and Laos refers to the Lao People's Democratic Republic.


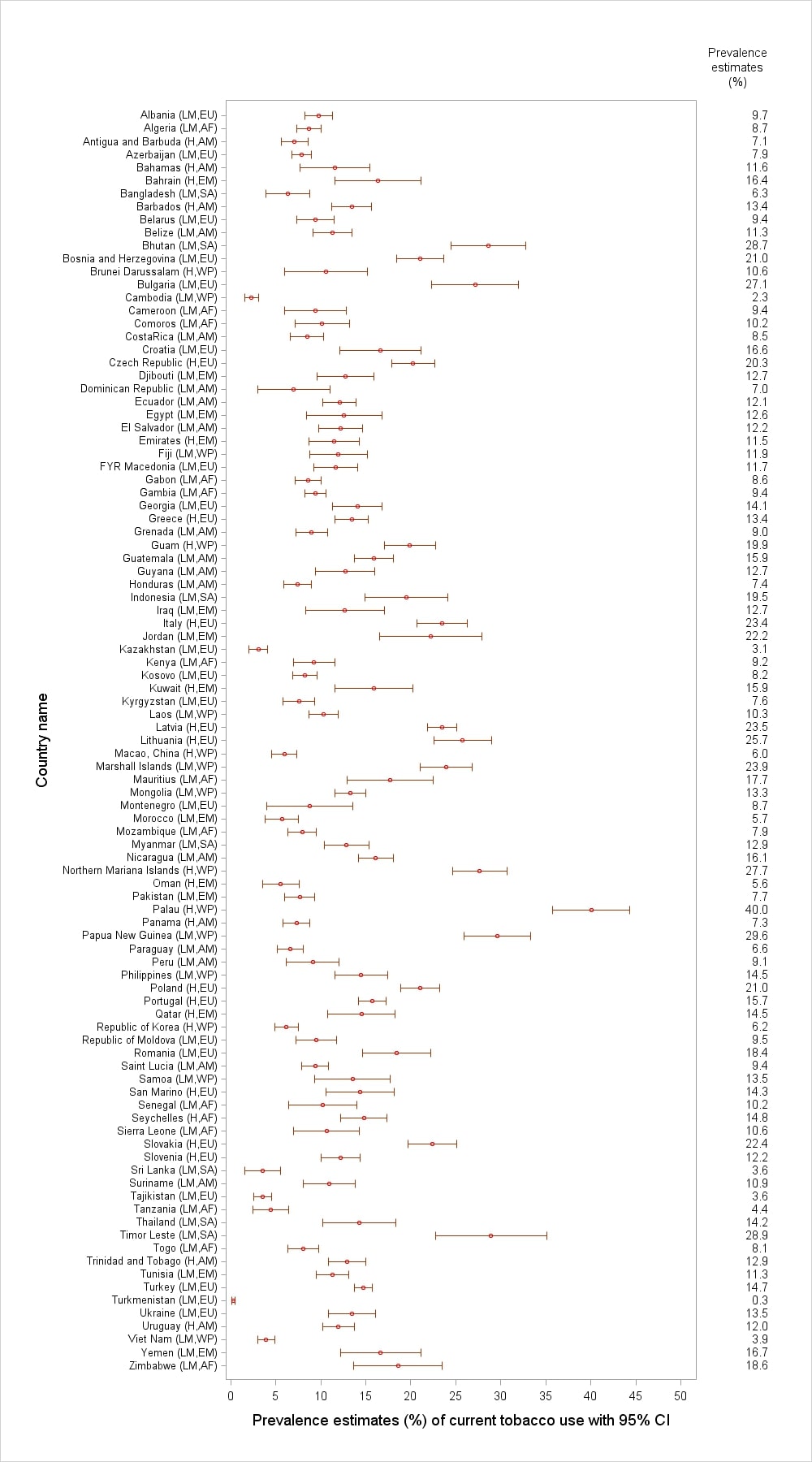

Supplement: S1 Fig — (DOCX) [file pgph.0000060.s001.docx]
