# Supplementary material for: Machine learning models of tobacco susceptibility and current use among adolescents from 97 countries in the Global Youth Tobacco Survey, 2013-2017
Source: PLOS Glob Public Health. 2021 Dec 8;1(12):e0000060. doi: 10.1371/journal.pgph.0000060 (PMC10021689; doi:10.1371/journal.pgph.0000060)
Supplement: S1 Table — (DOCX) [file pgph.0000060.s002.docx]

**S1 Table**

**Table A. Outcome and predictor variables, Global Youth Tobacco Survey**

^a^ GYTS respondents who responded to “No” for all three questions were identified as tobacco-naïve adolescents and included in regression models of susceptibility to tobacco use. “No” responses were coded as “0” and “Yes” responses as “1” for statistical analyses. To measure current tobacco use, Italy surveyed one question of smoking cigarettes and Turkey surveyed two questions of smoking cigarettes and using smoked tobacco products other than cigarettes in their GYTS questionnaire.

| **Variables** | **GYTS items** | **Item responses** | **Response coding** |
| --- | --- | --- | --- |
| ***Outcome variables*** | | | |
| Current tobacco use | 1. During the past 30 days, on how many days did you smoke cigarettes? | a. 0 days  b. 1 or 2 days  c. 3 to 5 days  d. 6 to 9 days  e. 10 to 19 days  f. 20 to 29 days  g. All 30 days | No = “0 days” for a question of smoking cigarettes and “No” for questions of smoked tobacco and smokeless tobacco products use  Yes = “1 to all 30 days” for a question of smoking cigarette or “Yes” for any of the other two questions |
|  | 2. During the past 30 days, did you use any form of smoked tobacco products other than cigarettes? | a. Yes  b. No |  |
|  | 3. During the past 30 days, did you use any form of smokeless tobacco products? | a. Yes  b. No |  |
| Tobacco-naïve adolescents | 1. Have you ever tried or experimented with cigarette smoking, even one or two puffs? | a. Yes  b. No | No = “No” for all three questions^a^  Yes = “Yes” for any of the three questions |
|  | 2. Have you ever tried or experimented with any form of smoked tobacco products other than cigarettes? | a. Yes  b. No |  |
|  | 3. Have you ever tried or experimented with any form of smokeless tobacco products? | a. Yes  b. No |  |
| Susceptibility to tobacco use | 1. If one of your best friends offered you a tobacco product, would you use it?  2. At any time during the next 12 months, do you think you will use any form of tobacco? | a. Definitely not  b. Probably not  c. Probably yes  d. Definitely yes | No = “Definitely not” for both questions  Yes = any other responses for either one of the two questions |
| ***Predictor variables*** | | | |
| Age in years | How old are you? | a. 11 years old or younger  b. 12 years old  c. 13 years old  d. 14 years old  e. 15 years old  f. 16 years old  g. 17 years old or older | 1 = 13 years old  2 = 14 years old  3 = 15 years old |
| Sex | What is your sex? | a. Male  b. Female | 1 = Male  0 = Female |
| Exposure to secondhand smoke (SHS) inside home | During the past 7 days, on how many days has anyone smoked inside your home, in your presence? | a. 0 days  b. 1 to 2 days  c. 3 to 4 days  d. 5 to 6 days  e. 7 days | No = 0 days  Yes = 1 day to 7 days |
| Exposure to SHS indoor public places | During the past 7 days, on how many days has anyone smoked in your presence, inside any enclosed public place, other than your home (such as: school, shops, restaurants, shopping malls, movie theaters)? | a. 0 days  b. 1 to 2 days  c. 3 to 4 days  d. 5 to 6 days  e. 7 days | No = 0 days  Yes = 1 day to 7 days |
| Exposure to SHS outdoor public places | During the past 7 days, on how many days has anyone smoked in your presence, at any outdoor public place (such as: playgrounds, sidewalks, entrances to buildings, parks, beaches)? | a. 0 days  b. 1 to 2 days  c. 3 to 4 days  d. 5 to 6 days  e. 7 days | No = 0 days  Yes = 1 day to 7 days |
| Exposure to SHS inside or outside school | During the past 30 days, did you see anyone smoke inside the school building or outside on school property? | a. Yes  b. No | No = No  Yes = Yes |
| Knowledge about harmful  effects of SHS | Do you think the smoke from other people’s tobacco smoking is harmful to you? | a. Definitely not  b. Probably not  c. Probably yes  d. Definitely yes | No = Definitely not  Yes = any other responses |
| Support for indoor smoke-free air policy (SFP) | Are you in favor of banning smoking inside enclosed public places (such as: schools, shops, restaurants, shopping malls, movie theaters)? | a. Yes  b. No | No = No  Yes = Yes |
| Support for outdoor SFP | Are you in favor of banning smoking at outdoor public places (such as: playgrounds, sidewalks, entrances to buildings, parks, beaches)? | a. Yes  b. No | No = No  Yes = Yes |
| Exposure to anti-tobacco media messages | During the past 30 days, did you see or hear any anti-tobacco media messages on television, radio, internet, billboards, posters, newspapers, magazines, or movies? | a. Yes  b. No | No = No  Yes = Yes |
| Exposure to anti-tobacco messages at social events | During the past 30 days, did you see or hear any anti-tobacco messages at sports events, fairs, concerts, or community events, or social gatherings? | a. I did not go to sports events, fairs, concerts, or community events, or social gatherings in the past 30 days  b. Yes  c. No | No = No or I did not go to sports events, fairs, concerts, or community events, or social gatherings in the past 30 days  Yes = Yes |
| Exposure to health warning messages on cigarette packages | During the past 30 days, did you see any health warnings on cigarette packages? | a. Yes, but I didn’t think much of them  b. Yes, and they led me to think about quitting smoking or not starting smoking  c. No | No = No  Yes = any other responses |
| Exposure to anti-smoking school education | During the past 12 months, were you taught in any of your classes about the dangers of tobacco use? | a. Yes  b. No  c. I don’t know | No = No or I do not know  Yes = Yes |
| Exposure to pro-tobacco media advertisements | During the past 30 days, did you see any people using tobacco on TV, in videos, or in movies? | a. I did not watch TV, videos, or movies in the past 30 days  b. Yes  c. No | No = No or I did not watch TV, videos, or movies in the past 30 days  Yes = Yes |
| Exposure to point-of-sale (POS) pro-tobacco advertisements or promotions | During the past 30 days, did you see any advertisements or promotions for tobacco  products at points of sale (such as: stores, shops, kiosks, etc.)? | a. I did not visit any points of sale in the past 30 days  b. Yes  c. No | No = No or I did not visit any points of sale in the past 30 days  Yes = Yes |
| Exposure/receptivity to tobacco industry promotions | 1. Would you ever use or wear something that has a tobacco company or tobacco product name or picture on it such as a lighter, t-shirt, hat, or sunglasses? | a. Yes  b. Maybe  c. No | No = “No” for all three questions  Yes = “Yes” or “Maybe” for any of the three questions |
|  | 2. Do you have something (for example, t-shirt, pen, backpack) with a tobacco product brand logo on it? | a. Yes  b. No |  |
|  | 3. Has a person working for a tobacco company ever offered you a free tobacco product? | a. Yes  b. No |  |

**Table B . Country-specific prevalence estimates (%, standard error of percentage) of susceptibility to tobacco use among tobacco-naïve adolescents and use of any tobacco product in the past 30 days among adolescents, aged 13-15 years, from 97 countries in the Global Youth Tobacco Survey, 2013-2017.**

SEP=standard error of percent. In the parentheses next to country name, the first code indicates country-income level category (LM=low- and middle-income, H=high-income) and the second code indicates WHO region category (AF=Africa, AM=The Americas, EM=Eastern Mediterranean, EU=Europe, SA=Southeast Asia, WP=Western Pacific). FYR Macedonia refers to as the former Yugoslav Republic of Macedonia and Laos refers to as the Lao People's Democratic Republic.

|  | **Susceptibility to tobacco use among tobacco-naïve adolescents** | | **Current tobacco use**  **(used any product in past 30 days)** | |
| --- | --- | --- | --- | --- |
| **Country name** | **Weighted N** | **% (SEP)** | **Weighted N** | **% (SEP)** |
| Albania (LM,EU) | 12260 | 15.6 (0.9) | 12569 | 9.7 (0.7) |
| Algeria (LM,AF) | 37349 | 5.4 (0.4) | 79068 | 8.7 (0.7) |
| Antigua and Barbuda (H,AM) | 289 | 18.5 (1.3) | 164 | 7.1 (0.7) |
| Azerbaijan (LM,EU) | 16531 | 7.6 (0.8) | 22597 | 7.9 (0.5) |
| Bahamas (H,AM) | 2554 | 32.5 (9.9) | 1452 | 11.6 (1.9) |
| Bahrain (H,EM) | 4355 | 18.0 (1.4) | 6691 | 16.4 (2.4) |
| Bangladesh (LM,SA) | 474495 | 9.9 (1.7) | 396070 | 6.3 (1.2) |
| Barbados (H,AM) | 1194 | 24.8 (1.8) | 1082 | 13.4 (1.1) |
| Belarus (LM,EU) | 8032 | 6.5 (0.6) | 18715 | 9.4 (1.0) |
| Belize (LM,AM) | 2237 | 21.7 (1.6) | 1798 | 11.3 (1.0) |
| Bhutan (LM,SA) | 187 | 6.7 (0.7) | 1595 | 28.7 (1.0) |
| Bosnia and Herzegovina (LM,EU) | 4185 | 14.2 (1.2) | 14414 | 21.0 (1.3) |
| Brunei Darussalam (H,WP) | 1423 | 9.2 (1.2) | 2244 | 10.6 (2.1) |
| Bulgaria (LM,EU) | 13105 | 20.2 (1.3) | 42258 | 27.1 (2.3) |
| Cambodia (LM,WP) | 20147 | 5.6 (0.7) | 9482 | 2.3 (0.4) |
| Cameroon (LM,AF) | 116275 | 26.9 (3.6) | 58198 | 9.4 (1.6) |
| Comoros (LM,AF) | 3378 | 28.3 (2.2) | 1757 | 10.2 (1.4) |
| Costa Rica (LM,AM) | 21453 | 15.2 (1.3) | 17446 | 8.5 (0.9) |
| Croatia (LM,EU) | 9429 | 17.4 (1.2) | 18028 | 16.6 (2.2) |
| Czech Republic (H,EU) | 23841 | 22.5 (1.2) | 49220 | 20.3 (1.1) |
| Djibouti (LM,EM) | 3325 | 32.0 (3.1) | 2198 | 12.7 (1.5) |
| Dominican Republic (LM,AM) | 72476 | 16.7 (2.5) | 43676 | 7.0 (1.9) |
| Ecuador (LM,AM) | 77512 | 17.0 (1.3) | 88454 | 12.1 (0.9) |
| Egypt (LM,EM) | 301609 | 13.3 (2.6) | 437972 | 12.6 (2.1) |
| El Salvador (LM,AM) | 24283 | 12.5 (0.9) | 37888 | 12.2(1.2) |
| Emirates (H,EM) | 12671 | 16.3 (1.2) | 13880 | 11.5 (1.4) |
| Fiji (LM,WP) | 5418 | 20.1 (1.9) | 4827 | 11.9 (1.5) |
| FYR Macedonia (LM,EU) | 4828 | 14.0 (0.7) | 6415 | 11.7 (1.2) |
| Gabon (LM,AF) | 5091 | 16.0 (2.3) | 4238 | 8.6 (0.7) |
| Gambia (LM,AF) | 7620 | 22.6 (1.6) | 4747 | 9.4 (0.6) |
| Georgia (LM,EU) | 10915 | 15.1 (1.9) | 17965 | 14.1 (1.3) |
| Greece (H,EU) | 22603 | 12.9 (0.6) | 38448 | 13.4 (0.9) |
| Grenada (LM,AM) | 448 | 18.2 (1.4) | 348 | 9.0 (0.9) |
| Guam (H,WP) | 765 | 22.2 (2.5) | 1435 | 19.9 (1.4) |
| Guatemala (LM,AM) | 42888 | 15.2 (1.5) | 80180 | 15.9 (1.1) |
| Guyana (LM,AM) | 2523 | 16.5 (2.2) | 3105 | 12.7 (1.5) |
| Honduras (LM,AM) | 22515 | 13.0 (1.1) | 17021 | 7.4 (0.8) |
| Indonesia (LM,SA) | 400837 | 8.8 (0.8) | 1384111 | 19.5 (2.3) |
| Iraq (LM,EM) | 56064 | 16.3 (1.7) | 73050 | 12.7 (2.0) |
| Italy (H,EU) | 5638 | 33.3 (2.2) | 7316 | 23.4 (1.4) |
| Jordan (LM,EM) | 30064 | 18.2 (1.2) | 69795 | 22.2 (2.8) |
| Kazakhstan (LM,EU) | 9433 | 5.8 (0.9) | 6037 | 3.1 (0.5) |
| Kenya (LM,AF) | 214469 | 23.4 (2.0) | 122329 | 9.2 (1.1) |
| Kosovo (LM,EU) | 4470 | 9.2 (0.7) | 6075 | 8.2 (0.7) |
| Kuwait (H,EM) | 8375 | 20.0 (1.3) | 10698 | 15.9 (2.1) |
| Kyrgyzstan (LM,EU) | 6816 | 4.8 (0.6) | 14615 | 7.6 (0.9) |
| Laos (LM,WP) | 16514 | 9.5 (0.8) | 24387 | 10.3 (0.8) |
| Latvia (H,EU) | 2683 | 18.5 (1.4) | 10859 | 23.5 (0.8) |
| Lithuania (H,EU) | 4427 | 17.9 (1.9) | 22393 | 25.7 (1.6) |
| Macao, China (H,WP) | 1099 | 12.4 (2.2) | 701 | 6.0 (0.7) |
| Marshall Islands (LM,WP) | 229 | 28.3 (1.8) | 404 | 23.9 (1.5) |
| Mauritius (LM,AF) | 2593 | 10.4 (1.2) | 7290 | 17.7 (2.4) |
| Mongolia (LM,WP) | 16934 | 25.3 (1.1) | 17173 | 13.3 (0.8) |
| Montenegro (LM,EU) | 1665 | 11.3 (0.8) | 2661 | 8.7 (2.3) |
| Morocco (LM,EM) | 61452 | 6.8 (0.6) | 64602 | 5.7 (0.9) |
| Mozambique (LM,AF) | 82072 | 32.1 (1.4) | 28176 | 7.9 (0.8) |
| Myanmar (LM,SA) | 36922 | 5.9 (0.6) | 120140 | 12.9 (1.2) |
| Nicaragua (LM,AM) | 9667 | 15.7 (1.1) | 17949 | 16.1 (1.0) |
| Northern Mariana Islands (H,WP) | 145 | 14.3 (1.7) | 605 | 27.7 (1.5) |
| Oman (H,EM) | 8160 | 11.8 (1.8) | 5116 | 5.6 (0.9) |
| Pakistan (LM,EM) | 125391 | 10.1 (1.1) | 133357 | 7.7 (0.8) |
| Palau (H,WP) | 30 | 15.1 (2.5) | 277 | 40.0 (2.1) |
| Panama (H,AM) | 19016 | 16.3 (1.3) | 11242 | 7.3 (0.7) |
| Papua New Guinea (LM,WP) | 1239 | 18.0 (2.9) | 4420 | 29.6 (1.7) |
| Paraguay (LM,AM) | 25000 | 12.7 (1.1) | 17093 | 6.6 (0.7) |
| Peru (LM,AM) | 226171 | 23.2 (1.2) | 131146 | 9.1 (1.4) |
| Philippines (LM,WP) | 312151 | 13.1 (0.8) | 565482 | 14.5 (1.5) |
| Poland (H,EU) | 98120 | 27.2 (1.2) | 162894 | 21.0 (1.1) |
| Portugal (H,EU) | 22330 | 22.6 (0.7) | 25501 | 15.7 (0.8) |
| Qatar (H,EM) | 2218 | 21.9 (2.3) | 2443 | 14.5 (1.7) |
| Republic of Korea (H,WP) | 141883 | 12.3 (0.8) | 92436 | 6.2 (0.6) |
| Republic of Moldova (LM,EU) | 5857 | 10.4 (1.0) | 8608 | 9.5 (1.1) |
| Romania (LM,EU) | 40435 | 14.7 (1.1) | 86779 | 18.4 (1.9) |
| Saint Lucia (LM,AM) | 771 | 22.2 (1.5) | 523 | 9.4 (0.7) |
| Samoa (LM,WP) | 1196 | 16.5 (1.9) | 1477 | 13.5 (2.0) |
| San Marino (H,EU) | 132 | 35.6 (2.4) | 80 | 14.3 (1.9) |
| Senegal (LM,AF) | 107130 | 26.2 (4.4) | 57340 | 10.2 (1.8) |
| Seychelles (H,AF) | 346 | 17.4 (1.7) | 601 | 14.8 (1.3) |
| Sierra Leone (LM,AF) | 20884 | 25.8 (2.4) | 13452 | 10.6 (1.8) |
| Slovakia (H,EU) | 10653 | 20.3 (1.3) | 27620 | 22.4 (1.3) |
| Slovenia (H,EU) | 4559 | 17.8 (1.3) | 5204 | 12.2 (1.0) |
| Sri Lanka (LM,SA) | 18687 | 2.5 (0.5) | 30855 | 3.6 (0.9) |
| Suriname (LM,AM) | 1731 | 16.0 (1.0) | 1888 | 10.9 (1.3) |
| Tajikistan (LM,EU) | 19116 | 7.2 (0.9) | 12275 | 3.6 (0.5) |
| Tanzania (LM,AF) | 98912 | 6.5 (1.1) | 83777 | 4.4 (1.0) |
| Thailand (LM,SA) | 96187 | 7.4 (0.9) | 295720 | 14.2 (1.9) |
| Timor-Leste (LM,SA) | 5540 | 26.9 (3.3) | 17916 | 28.9 (2.9) |
| Togo (LM,AF) | 23804 | 15.2 (1.8) | 16585 | 8.1 (0.8) |
| Trinidad and Tobago (H,AM) | 4436 | 17.0 (1.4) | 5739 | 12.9 (1.0) |
| Tunisia (LM,EM) | 20589 | 9.5 (0.7) | 39040 | 11.3 (0.9) |
| Turkey (LM,EU) | 160330 | 9.7 (0.3) | 474342 | 14.7 (0.5) |
| Turkmenistan (LM,EU) | 1919 | 0.9 (0.2) | 656 | 0.3 (0.1) |
| Ukraine (LM,EU) | 61662 | 18.6 (2.8) | 91549 | 13.5 (1.3) |
| Uruguay (H,AM) | 12241 | 16.4 (1.0) | 14101 | 12.0 (0.9) |
| Viet Nam (LM,WP) | 54702 | 6.6 (0.7) | 38768 | 3.9 (0.5) |
| Yemen (LM,EM) | 54603 | 15.9 (2.3) | 115454 | 16.7 (2.1) |
| Zimbabwe (LM,AF) | 28944 | 36.6 (2.0) | 32285 | 18.6 (2.4) |

**Table C. Prevalence estimates of use of cigarettes, smoked tobacco products other than cigarettes, and smokless tobacco products among adolescents, aged 13-15 years, from 97 countries in the Global Youth Tobacco Survey, 2013-2017, by WHO region and country-income level.**

^a^ Weighted percentages and, in parentheses, weighted counts. CI=confidence interval.

^***^*p*<0.0001, ^*^*p*<0.01, ^*^*p*<0.05

|  | Cigarettes | | Smoked tobacco products other than cigarettes | | Smokeless tobacco products | |
| --- | --- | --- | --- | --- | --- | --- |
| Region and income groups | % (N)^a^ | 95% CI | % (N)^a^ | 95% CI | % (N)^a^ | 95% CI |
| WHO region |  |  |  |  |  |  |
| Africa (n=14) | 4.1 (247,585) | 3.5-4.8 | 2.7 (204,706) | 2.7-4.0 | 3.3 (203,877) | 2.9-3.8 |
| The Americas (n=20) | 7.3 (335,353) | 6.4-8.1 | 3.7 (208,140) | 3.7-5.1 | 2.2 (103,893) | 1.9-2.5 |
| Eastern Mediterranean (n=13) | 4.7 (380,954) | 3.5-5.8 | 4.4 (486,175) | 4.4-7.2 | 3.8 (316,692) | 2.8-4.8 |
| Europe (n=28) | 8.7 (709,455) | 8.3-9.2 | 7.8 (643,683) | 7.8-8.9 | 3.4 (170,713) | 3.0-3.7 |
| Southeast Asia (n=7) | 10.3 (1,694,929) | 8.4-12.2 | 2.3 (487,011) | 2.3-3.4 | 3.3 (561,217) | 2.5-4.1 |
| Western Pacific (n=15) | 8.2 (563,363) | 7.0-9.3 | 1.7 (180,096) | 1.7-3.4 | 2.0 (143,741) | 1.5-2.6 |
| Across WHO region χ^2^ | 2479.0^***^ |  | 3133.9^***^ |  | 302.6^**^ |  |
| Country-income |  |  |  |  |  |  |
| High income (n=28) | 9.4 (367,337) | 8.7-10.2 | 4.6 (199,395) | 4.6-5.6 | 2.8 (108,765) | 2.3-3.2 |
| Low and middle income (n=69) | 7.7 (3,564,302) | 7.0-8.4 | 3.9 (2,010,414) | 3.9-4.7 | 3.1 (1,391,366) | 2.7-3.5 |
| Across country-income χ^2^ | 98.4^**^ |  | 39.5^**^ |  | 8.1 |  |
| *Total (n=97)* | 7.8 (3,931,639) | 7.1-8.5 | 4.3 (2,209,809) | 4.0-4.7 | 3.1 (1,500,132) | 2.7-3.5 |
